# Pyrogallol-Phloroglucinol-6 6-Bieckol on Attenuates High-Fat Diet-Induced Hypertension by Modulating Endothelial-to-Mesenchymal Transition in the Aorta of Mice

**DOI:** 10.1155/2021/8869085

**Published:** 2021-01-26

**Authors:** Myeongjoo Son, Seyeon Oh, Ji Tae Jang, Kuk Hui Son, Kyunghee Byun

**Affiliations:** ^1^Department of Anatomy & Cell Biology, Gachon University College of Medicine, Incheon 21936, Republic of Korea; ^2^Functional Cellular Networks Laboratory, College of Medicine, Department of Medicine, Graduate School and Lee Gil Ya Cancer and Diabetes Institute, Gachon University, Incheon 21999, Republic of Korea; ^3^Aqua Green Technology Co., Ltd., Smart Bldg., Jeju Science Park, Cheomdan-ro, Jeju 63243, Republic of Korea; ^4^Department of Thoracic and Cardiovascular Surgery, Gachon University Gil Medical Center, Gachon University, Incheon 21565, Republic of Korea

## Abstract

Endothelial-to-mesenchymal transition (EndMT), which is involved in the development of various cardiovascular diseases, is induced by dyslipidemia or obesity. In dyslipidemia, the increased levels of oxidized low-density lipoproteins (oxLDL) upregulated the lectin-type oxidized LDL receptor 1 (Lox-1), which then upregulated the down signaling pathways of PKC-*α*/MMPs/TGF-*β*/SMAD2 or 3 and increased the EndMT. In this study, we investigated the effect of pyrogallol-phloroglucinol-6,6-bieckol (PPB), which is a compound of *Ecklonia cava* (*E. cava*), on decreased blood pressure (BP) by attenuating the EndMT in a high-fat diet- (HFD-) fed animal model. We also investigated PPB's attenuation effect on EndMT in oxLDL-treated mouse endothelial cells as an *in vitro* model. The results indicated that, in the aorta or endothelial cells of mice, the HFD or oxLDL treatment significantly increased the expression of Lox-1/PKC-*α*/MMP9/TGF-*β*/SMAD2/SMAD3. The PPB treatment significantly decreased its expression. In contrast, the HFD or oxLDL treatment significantly decreased the expression of the EC markers (PECAM-1 and vWF) while the PPB treatment significantly increased them. Moreover, the HFD or oxLDL treatment significantly increased the expression of the mesenchymal cell markers (*α*-SMA and vimentin) while PPB treatment significantly decreased them. PPB decreased the intima-media thickness and extracellular matrix amount of the aorta and attenuated the BP, which was increased by the HFD. In conclusion, PPB attenuated the upregulation of Lox-1/PKC-*α*/MMP9/TGF-*β*/SMAD2 and 3 and restored the EndMT in HFD-fed animals. Moreover, PPB showed a restoring effect on HFD-induced hypertension.

## 1. Introduction

Neointimal hyperplasia is the main pathophysiology of many cardiovascular diseases, such as atherosclerosis and hypertension [1]. Endothelial cell (EC) damage triggers intimal hyperplasia [2]. A disrupted EC layer of the intima of the arteries causes them to lose their ability to prevent underlying vascular smooth muscle cells (VSMCs) from migrating to the luminal side [2]. Exposing the blood elements, such as inflammatory cells, initiates VSMC proliferation, which causes neointimal hyperplasia [2]. In addition, EC could be a source of smooth, muscle-like cells migrating through the endothelial-to-mesenchymal transition (EndMT), which is involved in the neointimal hyperplasia processes [1, 3]. Through the EndMT, the ECs lose their cell-to-cell contact and cell polarity, which leads to morphological changes (e.g., spindle-shaped). These phenotypic changes are accompanied by increased cell migration or invasion or enhanced extracellular matrix production [4, 5]. EndMT also leads to molecular changes in the ECs. As the endothelial markers such as the vascular endothelial cadherin (VE-cadherin), von Willebrand factor (vWF), and platelet endothelial cell adhesion molecule-1 (PECAM-1 or CD31) are decreased, the mesenchymal markers such as alpha-smooth muscle actin (*α*-SMA), N-cadherin, vimentin, fibronectin, and calponin are increased [6, 7]. Many triggering factors, such as shear stress, hyperglycemia, dyslipidemia, and hyperhomocystinuria, induce EndMT [8, 9]. A high-fat diet (HFD) or obesity induces hypercholesterolemia or dyslipidemia, which leads to increased serum levels of oxidized, low-density lipoproteins (oxLDL) [10, 11].

Several studies showed that increased oxLDL levels might initiate the EndMT by inducing expression of the lectin-type oxidized LDL receptor-1 (Lox-1) [11–15]. ECs treated with oxLDL showed increased protein levels of transforming growth factor-beta (TGF-*β*) and Lox-1 [12–15]. Increased Lox-1 activity leads to the upregulation of the protein kinase C (PKC) signal pathway, which results in the overexpression of matrix metallopeptidases (MMPs) [16]. Increased PKC and MMP activity induce the activation of endogenous TGF-*β* [17]. The TGF-*β* superfamily consists of isoforms TGF-*β*1 and TGF-*β*2 and bone morphogenetic proteins [18]. In ECs, the TGF-*β* ligand binds to TGF*β*R2 and then activates TGF*β*R1 by phosphorylation [18]. The activated TGF*β*R1 activates SMAD2/3 leading to the translocation of SMADs to the nucleus, and then, the SMADs upregulate the expressions of the EndMT genes such as Snail, Twist, and ZEB [19].

Pyrogallol-phloroglucinol-6,6-bieckol (PPB), a compound of *Ecklonia cava* (*E. cava*), which is a brown alga, significantly inhibits monocyte migration and decreases monocyte-induced EC and VSMC dysfunction [20]. Also, PPB improves blood circulation by reducing the adhesion molecule expression, EC death, VSMC proliferation and migration, blood pressure (BP), and lipoprotein and cholesterol levels in a diet-induced obese model [21]. However, whether PPB modulates the EndMT, which is induced by hyperlipidemia, remains unclear. In this study, we evaluated the effect of PPB on EndMT induced by oxLDL in an in vitro model and EndMT induced by a HFD in an animal model. Moreover, we also evaluated the effect of PPB on the regulation of Lox-1/PKC/MMP9, which leads to the upregulation of TGF-*β*/SMAD2 and 3 and decreased HFD-induced BP.

## 2. Materials and Methods

### 2.1. Preparation of ECE and Isolation of PPB

The ECE was obtained from the Aqua Green Technology Co., Ltd. (Jeju, Korea). The *E. cava* raw material was thoroughly washed with pure water and air-dried at room temperature for 48 h. It was ground and added with 50% ethanol, followed by heating at 85°C for 12 h. The ECE were filtered and then, when concentrated, sterilized by heating at high temperature for 40–60 min and then spray-dried.

The PPB, which is one of the representative phlorotannins of *E. cava*, was then isolated as in the previous studies [20, 21]. Briefly, centrifugal partition chromatography was performed using a two-phase solvent system that mixed distilled water, ethyl acetate, methanol, and n-hexane mixture (ratio 7 : 7 : 3 : 2). The organic stationary phase was filled in the chromatography column. In contrast, the mobile phase was filled in the column in a descending manner at the flow rate (2 mL per min) and used for separation. Furthermore, the PPB purity was determined to be around 91.24%, which was used in the studies [20, 21].

### 2.2. HFD-Fed Animal Model

C57BL/6N male mice (8 weeks old) were bought from Orient Bio (Sungnam, Korea) and kept at a constant temperature of approximately 23°C–24°C, relative humidity of 50%, and light/dark cycle of 12 h/12 h. The mice were randomly divided into six groups (five mice per group):

(i)NFD-fed group, in which the mice were fed with a regular chow diet (PicoLab Rodent diet, Fort Worth, TX, USA) for four weeks and then orally administered with 0.9% normal saline for the last four weeks

(ii)HFD-fed group, in which the mice were fed with a 45% high-fat diet (Research Diet Inc., New Brunswick, NJ, USA) for four weeks and then orally administered with 0.9% normal saline for the last four weeks

(iii–v)HFD-fed group, in which the mice were fed with 45% high-fat diet for four weeks and then orally administered with E. cava extract (ECE) (iii) 50 mg/kg/day, (iv) 100 mg/kg/day, and (v) 150 mg/kg/day for the last four weeks

(vi)HFD-fed group, in which the mice were fed with a 45% high-fat diet for four weeks and then orally administered with 2.5 mg/kg/day PPB for the last four weeks

At the end of the eight weeks, all mice were sacrificed following the ethical principles of the Institutional Animal Care and Use Committee of Gachon University (approval number: LCDI-2019-0097). Body weight and fat weight were measured using by Minispec MQ Series (Bruker, MA, USA) before sacrifice, and total cholesterol, low-density lipoprotein (LDL), and triglycerides were measured at the time of sacrifice. All molecular experiments were carried out using HFD-fed 100 mg/kg/day ECE and PPB.

### 2.3. Immunofluorescence

Aorta tissue paraffin blocks were sectioned at 10 *μ*m, placed on gelatin-coating slides, and dried at 37°C for 48 h. The tissue slides were deparaffinized and then rinsed with phosphate-buffered saline (PBS); we incubated them with an animal serum to inhibit antibody nonspecific binding. Then, we incubated them with primary antibodies (anti-PECAM-1, anti-vWF, anti-*α*-SMA, anti-vimentin, and anti-collagen type *Ι* antibody) and rinsed them with PBS three times. The tissue slides were then exposed to the fluorescence conjugated secondary antibodies incubated for 1 h with a blocking solution and rinsed with PBS three times. Moreover, the rinsed tissues were then incubated for 5 min with 4′,6-diamidino-2-phenylindole (DAPI) solution, rinsed with PBS, and mounted with vector shield solution (Vector Laboratories). The fluorescence signal was detected using a confocal microscope (LSM 710, Carl Zeiss, Oberkochen, Germany).

### 2.4. Histological Analysis

To measure intima-media thickness of the aorta, the aorta tissue slides were stained with hematoxylin & eosin (HE). The tissue slides were deparaffinized using xylene followed by rehydration steps: 100%, 95%, 90%, 80%, and 70% of ethanol for 1 min of each step. After we rinsed the slides three times with water, we incubated them with a hematoxylin solution and washed them in running tap water for 3 min. The tissue slides were submerged into eosin solution for 1 min and then washed with water. Stained slides were mounted with DPX solution and visualized by light microscopy. Intima-media thickness of HE-stained aorta images was measured by ImageJ software (NIH, DC, USA) [22].

Masson's trichrome (MT) stain validates fibrosis of the aorta. The aorta tissue slides also were deparaffinized and rehydrated. Tissue slides were refixed with Bouin's solution for 24 hr and rinsed with running tap water for 3 min. The slides were submerged with Weigert's iron hematoxylin solution for 10 min and Biebrich scarlet-acid fuchsin solution for 15 min and then differentiated with phosphomolybdic-phosphotungstic acid solution for 10 min. Finally, slides were transferred to aniline blue solution for 3 min and then washed with water. Stained slides were mounted with DPX solution and visualized by light microscopy. Collagen appears with blue color in the fibrosis area, and vascular smooth muscle appears with red color. MT-stained aorta images were measured by ImageJ software.

### 2.5. RNA Extraction and Quantitative Real-Time Polymerase Chain Reaction (qRT-PCR)

The RNA from the cell sample or mouse sample was isolated using the RNAiso Plus reagent (TAKARA, Japan) following the provided instructions. Briefly, the pellet was resuspended with 1 mL of the RNAiso Plus reagent and mixed with 0.1 mL chloroform (Amresco, OH, USA) and then centrifuged at 14,000 × g for 20 min at 4°C. The supernatant was mixed with 0.25 mL 100% isopropyl alcohol, and the extracted RNA pellets were washed with 70% ethanol and centrifuged at 7,500 × g for 5 min at 4°C. The dried pellets were dissolved in 10–30 *μ*L diethylpyrocarbonate- (DEPC-) treated water, and RNA was quantified and checked using by Nanodrop 2000 (Thermo Fisher Scientific, MA, USA). Complementary DNA (cDNA) was prepared from RNA using a cDNA synthesis kit (PrimeScript™, TAKARA). A quantitative real-time polymerase chain reaction (qRT-PCR) determined the RNA levels. The used primers were mixed with distilled water and then placed in a 384-well qRT-PCR plate. Template (cDNA) and SYBR green (TAKARA) were subsequently added and then validated using a PCR machine (Bio-Rad, Hercules, CA, USA) [22]. Table S1 lists the genes of interest.

### 2.6. oxLDL-Treated In Vitro Model

SVEC4–10 cells (mouse vascular epithelium) were purchased from the Korean cell line bank (Seoul, Korea). Dulbecco's modified Eagle's medium (Gibco), 1% penicillin-streptomycin (Gibco), and 10% fetal bovine serum were used as a growth medium. The cells were then treated with PBS (PBS group) or oxLDL (5 *μ*g/mL; Thermo Fisher Scientific) plus PBS (oxLDL/PBS group), oxLDL plus ECE (oxLDL/ECE; 50 *μ*g/mL), or oxLDL plus PPB (oxLDL/PPB; 1.8 *μ*g/mL) for 24 h. The treated cells were rinsed with PBS and prepared for some experiments.

### 2.7. Immunocytochemistry (ICC)

The SVEC4–10 cells (10^4^ cells/well) were seeded in an 8-well plate (SPL Life Sciences, Korea). After we removed the cell growth medium and rinsed it with phosphate-buffered saline three times, we incubated it with serum for blocking and primary antibodies for 24 h (anti-PECAM-1, anti-vWF, anti-*α*-SMA, and anti-vimentin antibody) and rinsed it with PBS. The seed cells were incubated for 1 h with fluorescence conjugated secondary antibodies and then washed out with PBS. Moreover, the rinsed cells were then incubated for 3 min with DAPI solution to detect nuclei, rinsed with PBS, and mounted with vector shield solution. The fluorescence signal was detected using a confocal microscope (LSM 710).

### 2.8. Systolic Blood Pressure, Diastolic Blood Pressure, and Mean Arterial Blood Pressure Measurements

The systolic BP, diastolic BP, and mean arterial BP were measured using a noninvasive tail-cuff CODA system (Kent Scientific Corp., Torrington, CT, USA), as previously described [20–22]. The mouse sublimation was conducted for 10 min for three days, and BP was measured on the last day.

### 2.9. Protein Extract and Western Blotting

For protein isolation from SVEC4–10, the cells were scraped with RIPA lysis buffer containing proteinase and phosphatase inhibitor (EzRIPA; ATTO; Tokyo, Japan) and incubated for 15 min on an ice container. After centrifuging at 14,000 × g for 15 min, at 4°C, supernatants were collected in a new tube and protein concentrations were analyzed using a bicinchoninic acid assay kit (BCA kit; Thermo Fisher Scientific, Inc.; Waltham, MA, USA). To validate protein expression, western blotting was conducted. Equal amounts of lysate proteins were separated by 8 or 10% sodium dodecyl sulfate polyacrylamide gel electrophoresis. Then, proteins were transferred to polyvinylidene fluoride membranes, which were incubated with appropriate diluted primary antibodies (Table S2) at 4°C. The incubated membranes were washed with tris buffered saline (TBS) containing 0.1% Tween 20 three times and incubated with secondary antibodies for 3 h at room temperature. Membranes were developed by enhanced chemiluminescence using LAS-4000s (GE Healthcare; Chicago, IL, USA).

### 2.10. Angiogenesis Assay

#### 2.10.1. Transwell Migration Assay

The SVEC4–10 cells (10^4^ cells/well) were seeded into the upper chamber of a 24-well transwell plate (SPL Life Sciences, Korea) for 12 h, and then, growth medium was changed to starvation medium for 24 h. In the bottom chamber, new starvation medium with PBS (PBS group) or oxLDL (5 *μ*g/mL) plus PBS (oxLDL/PBS group), oxLDL plus ECE (oxLDL/ECE; 50 *μ*g/mL), or oxLDL plus PPB (oxLDL/PPB; 1.8 *μ*g/mL) was placed for 30 h. Migratory cells pass through an 8 *μ*m pore size membrane on the bottom side. Nonmigratory cells in the upper chamber were removed using a cotton swab, and migratory cells were stained with hematoxylin for 3 min and quantified.

#### 2.10.2. Wound Migration Assay

The SVEC4–10 cells (10^4^ cells/well) were seeded into a 24-well culture multiplate and cultured to confluence for 48 h and then FBS starved for 36 h using growth medium without FBS (starvation medium). One perpendicular scratch was made in each well using a 5 mL pipette tip, and the starvation medium was changed to a new starvation medium with PBS (PBS group) or oxLDL (5 *μ*g/mL) plus PBS (oxLDL/PBS group), oxLDL plus ECE (oxLDL/ECE; 50 *μ*g/mL), or oxLDL plus PPB (oxLDL/PPB; 1.8 *μ*g/mL) for 24 h. Images were taken 30 h later using an Axio Observer apparatus and analyzed using ImageJ software.

### 2.11. Statistical Analysis

The results were expressed as the mean ± standard deviation. The Kruskal-Wallis test was used to validate the differences among the groups, and the Mann-Whitney *U* test was used in the SPSS ver. 22 software completing post hoc comparisons. The significant differences are indicated as follows: an asterisk (^∗^) versus NFD/saline or PBS and sharp (#) versus HFD/saline or oxLDL/PBS.

## 3. Results

### 3.1. ECE and PPB Attenuated the Increased Body Weight and Dyslipidemia

**Figure 1:**
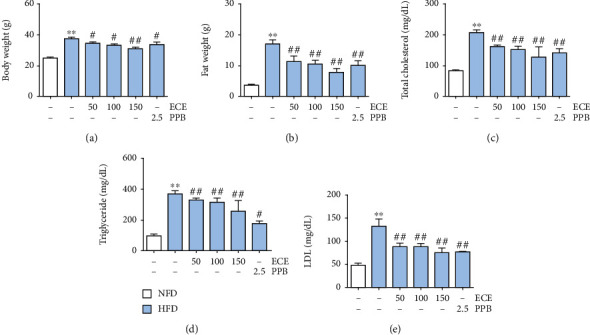
Comparative analysis of ECE and PPB administration on the reduction of body weight and dyslipidemia. (a) Body weight, (b) fat weight, (c) total cholesterol, (d) triglyceride, and (e) LDL were measured prior to sacrifice.

HFD significantly increased the body weight and fat weight in the HFD-fed group than in the NFD-fed group (Figures 1(a) and 1(b)). The body weight and fat weight of the ECE- or PPB-treated HFD-fed groups significantly decreased than those of the HFD-fed group. The decreasing effect of ECE or PPB on body weight and fat weight was most significant when ECE was administered at the level of 150 mg/kg/day. The total cholesterol level of HFD was significantly higher than that of the NFD-fed group (Figure 1(c)). The total cholesterol level decreased significantly after the administration of either ECE or PPB. However, there was no significant difference among the decreasing effects of the administration of ECE and PPB at the levels of 50, 100, and 150 mg/kg/day. Moreover, the triglyceride level increased significantly in the HFD-fed group over the NFD-fed group (Figure 1(d)). The increased level of triglyceride induced by HFD significantly decreased with the administration of either ECE or PPB. Also, the decreasing effect was most prominent at the level of 150 mg/kg/day of ECE and PPB. The LDL level of the HFD-fed group was significantly higher than that of the NFD-fed group (Figure 1(e)) and decreased by the administration of either ECE or PPB. Furthermore, the PPB administration had the most prominent effect on decreased LDL as compared with ECE.

### 3.2. ECE and PPB Attenuated the Expression of Lox-1 and PKC-*α* Induced by HFD or oxLDL Treatment

**Figure 2:**
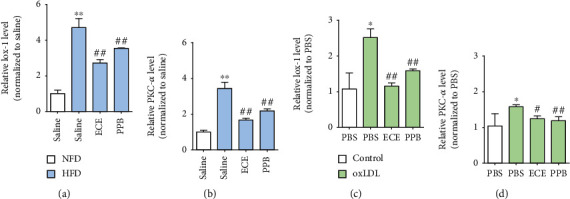
Comparative analysis of ECE and PPB treatment on the reduction of Lox-1 and PKC-*α* expression in HFD-fed mice and oxLDL-treated SVEC4–10 cells. mRNA levels of (a) Lox-1 and (b) PKC-*α* were determined in the aorta of HFD-fed mice using qRT-PCR. mRNA levels of (c) Lox-1 and (d) PKC-*α* were determined in oxLDL-treated SVEC4–10 cells using qRT-PCR. ^∗^vs. NFD/saline or PBS, ^#^vs. HFD/saline or oxLDL/PBS.

The Lox-1 expression level of the HFD-fed group in the aorta was significantly higher than that of the NFD-fed group. It decreased significantly after the administration of 100 mg/kg/day ECE or 2.5 mg/kg/day PPB (Figure 2(a)). Moreover, PKC-*α* expression in the aorta significantly increased in the HFD-fed group over that in the NFD-fed group. It decreased significantly with the administration of ECE or PPB (Figure 2(b)).

We made an in vitro model of dyslipidemia to mimic the environment with increased oxLDL levels induced by HFD by treating the SVEC4–10 cells, which are the ECs of mice with oxLDL. Through oxLDL treatment, the Lox-1 expression level in the SVEC4–10 cells increased significantly (Figure 2(c)), while it significantly decreased with the administration of either 50 *μ*g/mL ECE or 1.8 *μ*g/mL PPB. PKC-*α* expression significantly increased in the SVEC4–10 cells with the oxLDL treatment, while it significantly decreased with the administration of either ECE or PPB (Figure 2(d)).

### 3.3. ECE and PPB Attenuated the Expression of MMP9 and TGF-*β*/SMAD2/SMAD3 Induced by HFD or oxLDL Treatment

**Figure 3:**
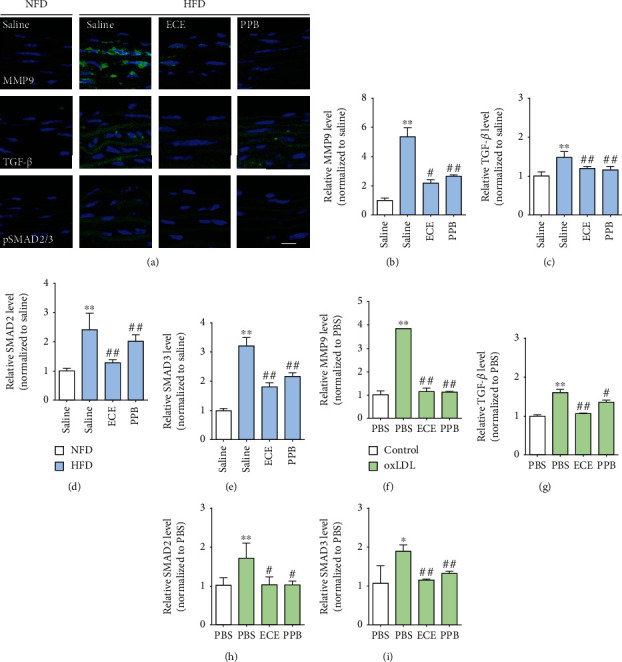
(a) Fluorescent images showing MMP9, TGF-*β*, and pSMAD2/3 expression in the aorta of HFD-fed mice. (b–e) mRNA levels of EndMT-related molecules (b) MMP9, (c) TGF-*β*, (d) SMAD2, and (e) SMAD3 were determined in HFD-fed mice using qRT-PCR. (f–i) mRNA levels of EndMT-related molecules (f) MMP9, (g) TGF-*β*, (h) SMAD2, and (i) SMAD3 were determined in oxLDL-treated SVEC4–10 cells using qRT-PCR. ^∗^vs. NFD/saline or PBS, ^#^vs. HFD/saline or oxLDL/PBS.

MMP9 expression in the aorta significantly increased in the HFD-fed group over that of the NFD-fed group. It significantly decreased with the administration of either 100 mg/kg/day ECE or 2.5 mg/kg/day PPB (Figures 3(a) and 3(b)). TGF-*β* expression in the aorta significantly increased in the HFD-fed group over that in the NFD-fed group. It decreased significantly with the administration of either ECE or PPB (Figures 3(a) and 3(c)). Moreover, SMAD2/3 expression in the aorta increased significantly in the HFD-fed group over that in the NFD-fed group. It decreased significantly with the administration of either ECE or PPB (Figures 3(d) and 3(e)). Phosphorylated SMAD2/3 expression in the aorta also increased significantly in the HFD-fed group over that in the NFD-fed group (Figure 3(a)), and it decreased significantly with the administration of either ECE or PPB.

MMP9 expression in the SVEC4–10 cells increased significantly with the oxLDL treatment and decreased significantly with the administration of either 50 *μ*g/mL ECE or 1.8 *μ*g/mL PPB (Figure 3(f) and Table S1). TGF-*β* expression in the SVEC4–10 cells increased significantly with the oxLDL treatment and decreased significantly with the administration of either 50 *μ*g/mL ECE or 1.8 *μ*g/mL PPB (Figure 3(g) and Table S1). SMAD2/3 expression in the SVEC4–10 cells increased significantly with the treatment of oxLDL and decreased significantly with the administration of either ECE or PPB (Figures 3(h) and 3(i) and Table S1).

### 3.4. ECE and PPB Attenuated the EndMT Induced by HFD or oxLDL Treatment

**Figure 4:**
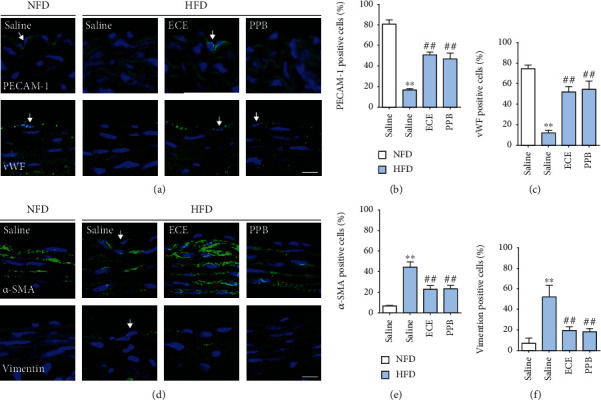
Comparative analysis of ECE and PPB administration on the reduction of EndMT in HFD-fed mice. (a) Confocal microscopic images demonstrating EC marker, PECAM-1 (arrow, top panel; green) and vWF (arrow, bottom panel; green), and nuclei (DAPI, blue) in the aorta of HFD-fed mice. Quantitative graphs demonstrating (b) the percentage of PECAM-1-positive cell (% of DAPI) and (c) vWF-positive cell (% of DAPI) from representative images. Scale bar = 10 *μ*m. (d) Confocal microscopic images demonstrating EndMT-related markers, *α*-SMA (arrow, top panel; green) and vimentin (arrow, bottom panel; green), and nuclei (DAPI, blue) in the aorta of HFD-fed mice. Scale bar = 10 *μ*m. Quantitative graphs demonstrating (e) the percentage of *α*-SMA-positive cell (% of DAPI) and (f) vimentin-positive cell (% of DAPI) from representative images. ^∗^vs. NFD/saline, ^#^vs. HFD/saline.

EndMT was evaluated by the decreased expression of the EC markers (PECAM-1 and vWF) and the increased expression of the mesenchymal cell markers (*α*-SMA and vimentin) in the HFD-fed mice. Each marker-positive cell ratio was presented as a ratio of each marker-positive cell to DAPI-positive cells of the intima in the aorta. The ratio of the PECAM-1-positive cells decreased significantly with the HFD treatment and returned with the administration of either 100 mg/kg/day ECE or 2.5 mg/kg/day PPB (Figures 4(a) and 4(b)). Moreover, the ratio of vWF-positive cells decreased significantly with the HFD treatment and returned with the administration of either ECE or PPB (Figures 4(a) and 4(c)).

The ratio of *α*-SMA-positive cells increased significantly with the HFD treatment and decreased significantly with the administration of either ECE or PPB (Figures 4(d) and 4(e)). The ratio of the vimentin-positive cells increased significantly with the HFD treatment and decreased significantly with the administration of either ECE or PPB (Figures 4(d) and 4(f)).

**Figure 5:**
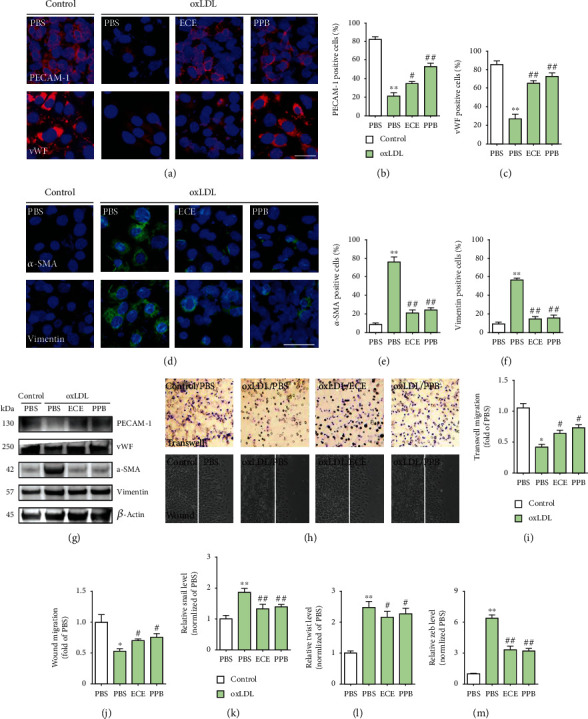
Comparative analysis of ECE and PPB treatment on the reduction of EndMT in oxLDL-treated SVEC4–10 cells. (a) Confocal fluorescence microscopic images demonstrating EC marker, PECAM-1 (top panel; red) and vWF (bottom panel; red), and nuclei (DAPI, blue) in oxLDL-treated SVEC4–10 cells. Quantitative graphs demonstrating (b) the percentage of PECAM-1-positive cells (% of DAPI) and (c) vWF-positive cells (% of DAPI) from representative images. Scale bar = 100 *μ*m. (d) Confocal fluorescence microscopic images demonstrating EndMT-related markers, *α*-SMA (top panel; green) and vimentin (bottom panel; green), and nuclei (DAPI, blue) in oxLDL-treated SVEC4–10 cells. Quantitative graphs demonstrating (e) the percentage of *α*-SMA-positive cells (% of DAPI) and (f) vimentin-positive cells (% of DAPI) from representative images. (g) Western blotting results demonstrating EC marker, PECAM-1 and vWF, and EndMT-related markers, *α*-SMA and vimentin. (h) Transwell migration assay and wound migration assay results showing endothelial property and quantitative graphs showing (i) moved cell to the opposite side or (j) moved cell from either side of the gap. Magnification: ×100. (k–m) mRNA levels of EndMT-related key factors (k) snail1, (l) twist1, and (m) zeb1 were determined in oxLDL-treated SVEC4–10 cells using qRT-PCR. ^∗^vs. PBS, ^#^vs. oxLDL/PBS.

The expressions of EndMT-related markers were also evaluated in the oxLDL-treated SVEC4–10 cells. The ratio of each marker-positive cell was presented as a ratio of each marker-positive cell to the DAPI-positive cell. The ratio of PECAM-1-positive cells was significantly decreased by the oxLDL treatment and restored by the administration of either 50 *μ*g/mL ECE or 1.8 *μ*g/mL PPB (Figures 5(a) and 5(b)). The ratio of the vWF-positive cell decreased significantly with the oxLDL treatment and returned with the administration of either ECE or PPB (Figures 5(a) and 5(c)).

The ratio of *α*-SMA-positive cells increased significantly with the oxLDL treatment and decreased significantly with the administration of either ECE or PPB (Figures 5(d) and 5(e)). The ratio of the vimentin-positive cells increased significantly with the oxLDL treatment and significantly decreased by the administration of either ECE or PPB (Figures 5(d) and 5(f)).

The endothelial property was measured by angiogenesis assays. The ratio of the migrating cell to the opposite side well decreased significantly with the oxLDL treatment and increased significantly with the administration of either ECE or PPB (Figures 5(h) and 5(i)), and the ratio of the migrating cell to either side of the gap also decreased significantly with the oxLDL treatment and increased significantly with the administration of either ECE or PPB (Figures 5(h) and 5(j)). In addition, EndMT-related key factors including snail, twist, and zeb1 expression in the SVEC4–10 cells increased significantly with the treatment of oxLDL and decreased significantly with the administration of either ECE or PPB (Figures 5(k)–5(m)).

### 3.5. ECE and PPB Attenuated the Increase of BP and Intima-Media Thickness of the Aorta Induced by HFD

**Figure 6:**
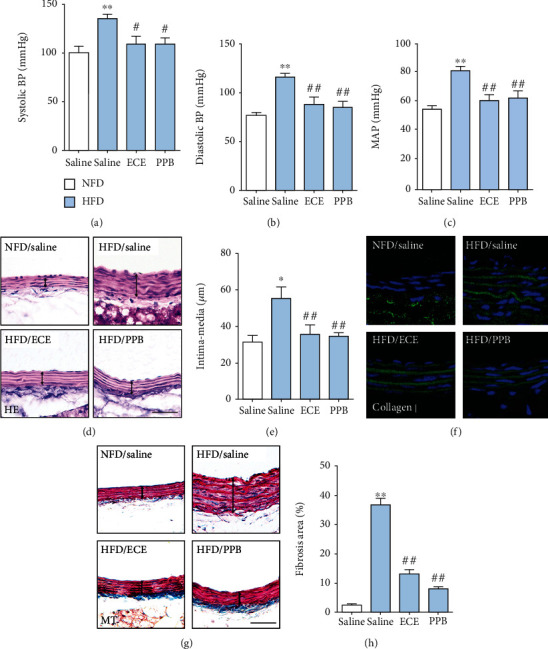
Comparative analysis of ECE and PPB administration on the regulation of blood pressure in HFD-fed animals. (a) Systolic and (b) diastolic blood pressures and (c) mean artery pressures (MAP) were measured prior to sacrifice. (d, e) Hematoxylin and eosin- (HE-) stained light microscopic images showing intima-media thickness of the aorta (black line) and the thickness acquired using representative HE-stained images. Scale bar = 50 *μ*m. (f) Immunofluorescent images showing collagen *Ι* (green, DAPI; blue) expression of the aorta and (g, h) Masson's trichrome- (MT-) stained light microscopic images showing fibrosis (blue) of the intima-media area of the aorta (black line) and the fibrosis area acquired using representative MT-stained images. Scale bar = 50 *μ*m. ^∗^vs. NFD/saline, ^#^vs. HFD/saline.

The systolic BP of HFD-fed mice was significantly higher than that of the NFD-fed mice. The increased BP induced by HFD decreased significantly with the administration of either ECE or PPB (Figure 6(a)). The diastolic BP increased significantly with HFD and decreased significantly with the administration of either ECE or PPB (Figure 6(b)). The mean artery pressures (MAP) increased significantly with the HFD treatment and decreased significantly with the administration of either ECE or PPB (Figure 6(c)).

The intima-media thickness of the aorta in the HFD-fed group significantly increased over that of the NFD-fed group. It decreased significantly with the administration of either ECE or PPB (Figures 6(d) and 6(e)). The amount of extracellular matrix, which was evaluated using collagen type *Ι* deposition and Masson's trichrome (MT) stain in the HFD-fed group, was significantly higher than that of the NFD-fed group and decreased significantly with the administration of either ECE or PPB (Figures 6(f)–6(h)).

## 4. Discussion

EndMT is a process in which ECs lose their features and acquired new characters as mesenchymal cells, which can cause endothelial dysfunction [8]. EndMT is involved in various cardiovascular diseases such as atherosclerosis, valvular disease, cardiac fibrosis, intimal hyperplasia, vein graft remodeling, and pulmonary hypertension [23]. Around 30% of ECs in the intima of the aorta underwent EndMT after four months of HFD treatment [24, 25]. Dyslipidemia induced by obesity or HFD results in increased circulating oxLDL levels, which lead to EndMT [26, 27]. oxLDL-treated human aortic ECs (HAECs) lead to decreased endothelial markers of E-cadherin and increased mesenchymal cell markers of N-cadherin and fibronectin [28]. Although many studies reported that oxLDL induces EndMT, related mechanisms remain unclear. Many studies suggested that Lox-1 is the main connection between oxLDL and EndMT [11, 29]. Binding oxLDL to Lox-1 upregulates TGF-*β* expression in ECs, increasing collagen production in myofibroblasts [11, 29]. When Lox-1 antibodies inhibited Lox-1, the oxLDL treatment could not increase the TGF-*β* secretion in the culture medium [15]. Thus, Lox-1 seems to play a role in the EndMT process, which is initiated by oxLDL.

In our study, HFD increased body weight and led to dyslipidemia, including increased total cholesterol, triglyceride, and LDL. Either ECE or PPB significantly attenuated the increased body weight and dyslipidemia induced by HFD. Lox-1 expression in the aorta increased significantly with HFD and decreased significantly with either ECE and PPB. Moreover, we made an in vitro model of dyslipidemia by treating mouse endothelial cells with oxLDL to evaluate whether oxLDL upregulates Lox-1 and ECE or PPB restored the upregulated Lox-1. Lox-1 expression in the SVEC4–10 cells increased significantly with the oxLDL treatment and decreased significantly with the administration of either ECE or PPB. Previous studies showed that oxLDL, Lox-1, and MMPs are colocalized in atherosclerotic lesions [30]. The interactions of oxLDL, Lox-1, and MMPs might be involved in the matrix breakdown in atherosclerotic plaques [31]. Atherosclerotic plaque disruption might be mediated by the Lox-1 upregulation of ECs, which was initiated by oxLDL [31].

oxLDL could upregulate the MMPs such as the membrane type 1-MMP (MT1-MMP), MMP1, MMP3, and MMP9 in ECs [16, 32, 33]. Also, PKC promotes the increase of MMPs by oxLDL [34]. MMPs also promote the degradation of collagen, elastin, and other matrix elements of the extracellular matrix and activate the inflammatory processes. Thus, those actions of MMPs are related to increased cardiovascular risk [35]. Moreover, MMP9 and MMP2 cleave the latent TGF-*β* by proteolysis and promote the activation of TGF-*β* [17].

In our study, PCK-*α* expression in the aorta increased significantly with HFD and decreased significantly with either ECE or PPB. PCK-*α* expression in the SVEC4–10 cells increased significantly with the oxLDL treatment and decreased significantly with the administration of either ECE or PPB. The expression levels of MMP9 and TGF-*β* in the aorta increased significantly with HFD and decreased significantly with either ECE or PPB. The expression of MMP9 and TGF-*β* in the SVEC4–10 cells increased significantly with the oxLDL treatment and decreased significantly with the administration of either ECE or PPB. It seems that either ECE or PPB was involved in downregulating the Lox-1/PCK-*α*/MMP9/TGF-*β* pathway, which was initiated by oxLDL. The decreasing effect on those signal pathways was more prominent when a higher dosage of ECE or PPB was administered. Among TGF-*β*'s various downsignaling pathways, the canonical TGF-*β* signal pathway, which regulates SMAD2/3, is the main driver of EndMT [36].

The activated TGF-*β* induced the phosphorylation of SMAD2/3, and the activated SMAD2/3 is translocated into the nucleus [37, 38]. The activated SMAD2/3 binds to SMAD-binding elements in the proximal promoters of the mesenchymal genes, such as Snail, Slug, and Twist1, and induces their expression [39, 40]. Snail, Slug, and Twist1 are the key factors of EndMT [39, 40]. The deficiency of Snail or Slug in EC prevents the induction of EndMT by TGF-*β*[41, 42]. In our study, SMAD2/3 expression in the aorta increased significantly with HFD and decreased significantly with either ECE or PPB. SMAD2/3 expression in the SVEC4–10 cells increased significantly with the oxLDL treatment and decreased significantly with the administration of either ECE or PPB. The expression of endothelial markers, such as PECAM-1 and vWF, in the intima of the aorta decreased significantly with HFD and increased significantly with either ECE or PPB. However, the expression of mesenchymal cell markers, such as *α*-SMA or vimentin, increased with HFD and decreased significantly with either ECE or PPB.

The expression of PECAM-1 and vWF in the SVEC4–10 cells decreased significantly with oxLDL treatment and increased significantly with either ECE or PPB. The increased expression of *α*-SMA or vimentin by oxLDL treatment decreased significantly with either ECE or PPB. It seems that either ECE or PPB had an attenuation effect on the EndMT in the aorta or SVEC4–10 cells, which was induced by dyslipidemia or oxLDL. The intima-media thickness of the arteries and arterial extracellular matrix is increased in hypertension [43]. During the EndMT process, ECs acquire the characteristics of mesenchymal stem-like cells and differentiate into multiple cell lineages, such as fibroblasts or myofibroblasts, which lead to extracellular matrix changes [44].

Through the proliferation of smooth muscle-like cells and increasing amounts of extracellular matrix proteins, the middle layer of the blood vessels is thickened and causes blood vessel remodeling [19]. The results of our study showed that BP was significantly increased by HFD and significantly decreased by either ECE or PPB. The intima-media thickness of the aorta increased significantly with HFD and decreased by either ECE or PPB. Moreover, the extracellular matrix amount of the aorta increased significantly with HFD and decreased significantly with either ECE or PPB. It seems that either ECE or PPB might potentially treat hypertension, especially that induced by obesity or dyslipidemia, by modulating EndMT.

It is known that disturbed oscillatory shear stress (OSS) upregulated Lox-1 expression in ECs [45]. We did not apply OSS to SVEC4–10 cells in our *in vitro* experiment; thus, our *in vitro* experiment could not fully replicate *in vivo* conditions in which ECs are exposed to OSS. However, our animal study showed that ECE or PPB downregulated Lox-1 expression which showed a similar pattern in the *in vitro* experiment.

## 5. Conclusions

Lox-1/PKC-*α*/MMP9 was upregulated by dyslipidemia, and those signal pathways increased TGF-*β* and SMAD2/3, which induced EndMT. Either ECE or PPB downregulated those signal pathways and decreased EndMT. Thus, ECE or PPB decreased the intima-media thickness and extracellular matrix amount in the aorta and attenuated the increased BP by HFD.

## Data Availability

All data supporting the conclusions of this article are included in this article.

## Supplementary Materials

Supplementary Materials Table S1: list of primers for quantitative real-time polymerase chain reaction (qRT-PCR). Table S2: list of antibodies for western blot. Figure S1: MMP9, TGF-*β*, pSMAD2, and pSMAD3 expression in oxLDL-treated SVEC4–10.
