# Supplementary material for: Pyrogallol-Phloroglucinol-6 6-Bieckol on Attenuates High-Fat Diet-Induced Hypertension by Modulating Endothelial-to-Mesenchymal Transition in the Aorta of Mice
Source: Oxid Med Cell Longev. 2021 Jan 26;2021:8869085. doi: 10.1155/2021/8869085 (PMC7857897; doi:10.1155/2021/8869085)
Supplement: Supplementary Materials — Table S1: list of primers for quantitative real-time polymerase chain reaction (qRT-PCR). Table S2: list of antibodies for western blot. Figure S1: MMP9, TGF-β, pSMAD2, and pSMAD3 expression in oxLDL-treated SVEC4–10. [file 8869085.f1.docx]

**Supplementary material**

**Table S1.** List of primer for quantitative real time polymerase chain reaction (qRT-PCR).

| **Gene name** | **Primer sequence** | |
| --- | --- | --- |
| *Actb* | Forward | 5’ – ACA AAG CTG TTC AGT GTC TCC A – 3’ |
|  | Reverse | 5’ – CTC CGT TTC CAG AAT ACA CAC A – 3’ |
| *Lox-1* | Forward | 5’ – CCT AAA GGT CTG CAT TTG CTT T – 3’ |
|  | Reverse | 5’ – TAA GGG TCA CTG ACA ACA CCA G – 3’ |
| *PKC-α* | Forward | 5’ – GAT CTC CAT CGG ACT GTT CTT C – 3’ |
|  | Reverse | 5’ – ATT TTG ATG TGC CCT TCT GAG T – 3’ |
| *MMP9* | Forward | 5’ – TCA CAC GAC ATC TTC CAG TAC C – 3’ |
|  | Reverse | 5’ – TGT CCA ATA GTC TGG TCA CAG G – 3’ |
| *TGF-β* | Forward | 5’ – CTG GCA GTA GCT CCC CTA TTT A – 3’ |
|  | Reverse | 5' – CAC CTC ATT TTG GAA ACT CAC A – 3’ |
| *SMAD2* | Forward | 5’ – GAA TGG ACA GGA AGA AAA GTG G – 3’ |
|  | Reverse | 5’ – CTC AAG CTC ATC TAA CCG TCC T – 3’ |
| *SMAD3* | Forward | 5’ – CGA GAA CAC TAA CTT CCC TGC T – 3’' |
|  | Reverse | 5’ – GGT CAC TGG TTT CTC CAT CTT C – 3’ |
| *Snail* | Forward | 5’ – AAG ATG CAC ATC CGA AGC CA– 3’ |
|  | Reverse | 5’ – CCT GCT GAG GCA TGG TTA CA– 3’ |
| *Twist* | Forward | 5’ – CTC GGA CAA GCT GAG CAA GA– 3’ |
|  | Reverse | 5’ – TCG TCA AAA AGT GGG GTG GG– 3’ |
| *Zeb* | Forward | 5’ – GGA GAA GCC TGC TCG TTC TT– 3’ |
|  | Reverse | 5’ – AAT GAC GGC GGT GTC TTG TT– 3’ |

**Table S2.** List of antibodies for western blot

| **Antibody** | **Company** | **Cat. No.** | **Antibody dilution** |
| --- | --- | --- | --- |
| β-actin | Cell signaling | #4967 | 1:1,000 |
| MMP9 | Gentex | GTX32891 | 1:500 |
| TGF-β | Abcam | Ab64715 | 1:1,000 |
| pSMAD2 | Cell signaling | #3108 | 1:1,000 |
| pSMAD3 | Cell signaling | #9520 | 1:1,000 |
| PECAM-1 | Santa cruz | SC-1506 | 1:100 |
| vWF | DAKO | A0082 | 1:200 |
| α-SMA | Invitrogen | 14-9760-82 | 1:100 |
| Vimentin | Santa cruz | SC-373717 | 1:100 |

**Figure S1.** MMP9, TGF-β, pSMAD2 and pSMAD3 expression in oxLDL treated SVEC4-10


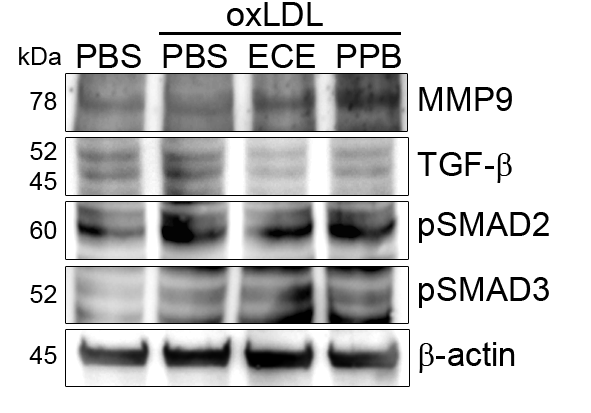


Western blotting results demonstrating MMP9 and TGF-β/pSMAD2/pSMAD3 expression in oxLDL treated SVEC4-10.
